# Membrane Binding of α-Synuclein Stimulates Expansion of SNARE-Dependent Fusion Pore

**DOI:** 10.3389/fcell.2021.663431

**Published:** 2021-07-19

**Authors:** Ryan Khounlo, Brenden J. D. Hawk, Tung-Mei Khu, Gyeongji Yoo, Nam Ki Lee, Josh Pierson, Yeon-Kyun Shin

**Affiliations:** ^1^Yeon-Kyun Shin Lab, Roy J. Carver Department of Biochemistry, Biophysics & Molecular Biology, Iowa State University, Ames, IA, United States; ^2^School of Interdisciplinary Bioscience and Bioengineering, Pohang University of Science and Technology, Pohang, South Korea; ^3^Department of Chemistry, Seoul National University, Seoul, South Korea

**Keywords:** SNARE, single-molecule, fusion pore, α-synuclein, TIRF

## Abstract

SNARE-dependent membrane fusion is essential for neurotransmitter release at the synapse. Recently, α-synuclein has emerged as an important regulator for membrane fusion. Misfolded α-synuclein oligomers are potent fusion inhibitors. However, the function of normal α-synuclein has been elusive. Here, we use the single vesicle-to-supported bilayer fusion assay to dissect the role of α-synuclein in membrane fusion. The assay employs 10 kD Rhodamine B-dextran as the content probe that can detect fusion pores larger than ∼6 nm. We find that the SNARE complex alone is inefficient at dilating fusion pores. However, α-synuclein dramatically increases the probability as well as the duration of large pores. When the SNARE-interacting C-terminal region of α-synuclein was truncated, the mutant behaves the same as the wild-type. However, the double proline mutants compromising membrane-binding show significantly reduced effects on fusion pore expansion. Thus, our results suggest that α-synuclein stimulates fusion pore expansion specifically through its membrane binding.

## Introduction

Communication between neurons, which underlies cognition, memory, and motor movement, is built upon neurotransmitter release at the synapse. In the neuron, cargo vesicles undergo membrane fusion with the plasma membrane, which releases the neurotransmitters into the synaptic cleft. It is established that the widely conserved SNARE (soluble N-ethylmaleimide sensitive factor attachment protein receptor) complex is the minimal machinery that drives membrane fusion (Sollner et al., 1993; Weber et al., 1998). SNARE motifs from vesicle-associated v-SNARE VAMP2 (or synaptobrevin 2) and those from target plasma membrane t-SNAREs, syntaxin-1A and SNAP-25, form a highly stable parallel coiled-coil (Poirier et al., 1998; Sutton et al., 1998). There is evidence that the SNARE complex zippers from the membrane distal region to the membrane-proximal region, culminating the folding energy toward apposition and merger of two membranes (Gao et al., 2012; Min et al., 2013; Shin et al., 2014).

**FIGURE 1:**
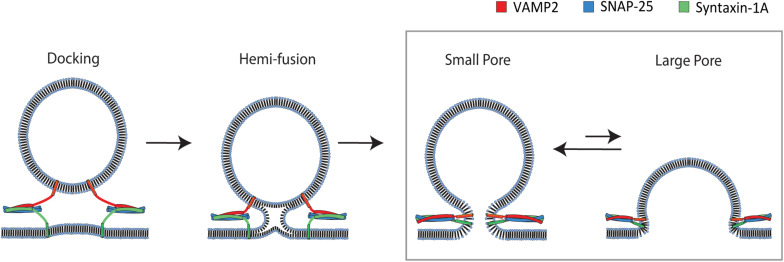
Pathway of SNARE-mediated membrane fusion. The v-SNARE VAMP2 located on the incoming vesicle initiates binding to the t-SNAREs syntaxin-1A and SNAP-25 on the plasma membrane. The v-and t-SNAREs begin to zipper into a coiled-coil that docks the vesicle to the plasma membrane. Continued zippering drives the outer leaflets of the two membranes to merge into a hemifusion state. Complete zippering drives the merger of the inner leaflets of two bilayers, which creates a small fusion pore. The small pore expands to a large pore, resulting in complete merger of the vesicle to the plasma membrane. The box shows the transition to which our single vesicle fusion assay is designed to be sensitive.

The membrane fusion process transits through distinct multiple stages (Figure 1). Hemifusion, in which outer leaflets of two bilayers are merged but inner leaflets are not (Lu et al., 2005; Xu et al., 2005), is followed by formation of a small aqueous fusion pore through which neurotransmitters are allowed to pass (Breckenridge and Almers, 1987; Han et al., 2004). The small pore then dilates to a large pore that could ultimately lead to a complete merger of two membranes into a single bilayer (Chernomordik and Kozlov, 2003). Alternatively, after a brief release, the fusion pore may close and the vesicle might then disengage from the plasma membrane without complete fusion, termed kiss-and-run (Alabi and Tsien, 2013). It is unknown what protein factors control the bias between two pathways.

α-synuclein (αS) is one of the most prevalent presynaptic proteins. But, when misfolded or aggregated, those aberrant forms are known to have close ties to the Parkinson’s disease and Lewy body dementia (Baba et al., 1998). Despite its abundance, its regular functions in the neuron have been elusive. Recently, there has been evidence that αS controls the size of the vesicle pool (Nemani et al., 2010), vesicle clustering (Diao et al., 2013), as well as vesicle docking to the plasma membrane (Lou et al., 2017). In addition, αS may stabilize SNARE complexes by interacting with VAMP2 (Sun et al., 2019).

Recently, Edwards and coworkers have proposed that αS plays a role in the dilation of the fusion pore, potentially biasing vesicle recycling toward the complete fusion pathway (Logan et al., 2017). They found that overexpressed αS promotes the release of the large cargo in chromaffin cells. However, it is unknown if αS also functions as a fusion pore dilator in the neuron and if it is the result of direct interaction with SNAREs or an indirect consequence of a multiprotein pathway.

On a molecular level, there are two well-known interactions for αS. The first is the interaction between its acidic C-terminal region and vesicle-attached VAMP2 (Burre et al., 2010). This specific interaction has been shown to be responsible for vesicle clustering (Diao et al., 2013), vesicle docking, as well as inhibition of vesicle fusion (Choi et al., 2013). The second is the membrane binding due to its affinity of the amphipathic N-terminal region toward negatively charged lipids (Jo et al., 2000). Despite extensive investigations, the physiological function of αS’s membrane binding is not fully understood (Snead and Eliezer, 2014).

In this work, we monitor the real-time dynamics of the fusion pore induced exclusively by SNARE proteins with the *in vitro* single vesicle-to-supported bilayer fusion assay (Liu et al., 2005; Kiessling et al., 2017; Kim and Shin, 2017; Kreutzberger et al., 2019). With this well-defined system, we intend to pinpoint the exact role that αS plays in the fusion pore dynamics. By encapsulating a fluorescent polymer probe of approximately 6 nm in diameter (Arrio-Dupont et al., 1996), we are able to observe the transient opening and contraction of the large fusion pore. We find that SNAREs alone are inefficient at generating a large fusion pore. When we include αS, however, we observe a dramatic increase in the number of vesicles that have the ability to open the large pore. In addition, we observe a significant increase of the duration of the large pore. Meanwhile, when the double proline mutants (A11P/V70P and T44P/A89P) of αS—which have reduced membrane binding—were used, the stimulating effects on fusion pore expansion were significantly diminished. In contrast, the truncation mutant in which VAMP2-interacting C-terminal region is deleted (αS 1-95), behaved the same as the wild-type.

## Results

### Single Vesicle-to-Supported Bilayer Fusion Assay to Monitor a Large Fusion Pore

**FIGURE 2:**
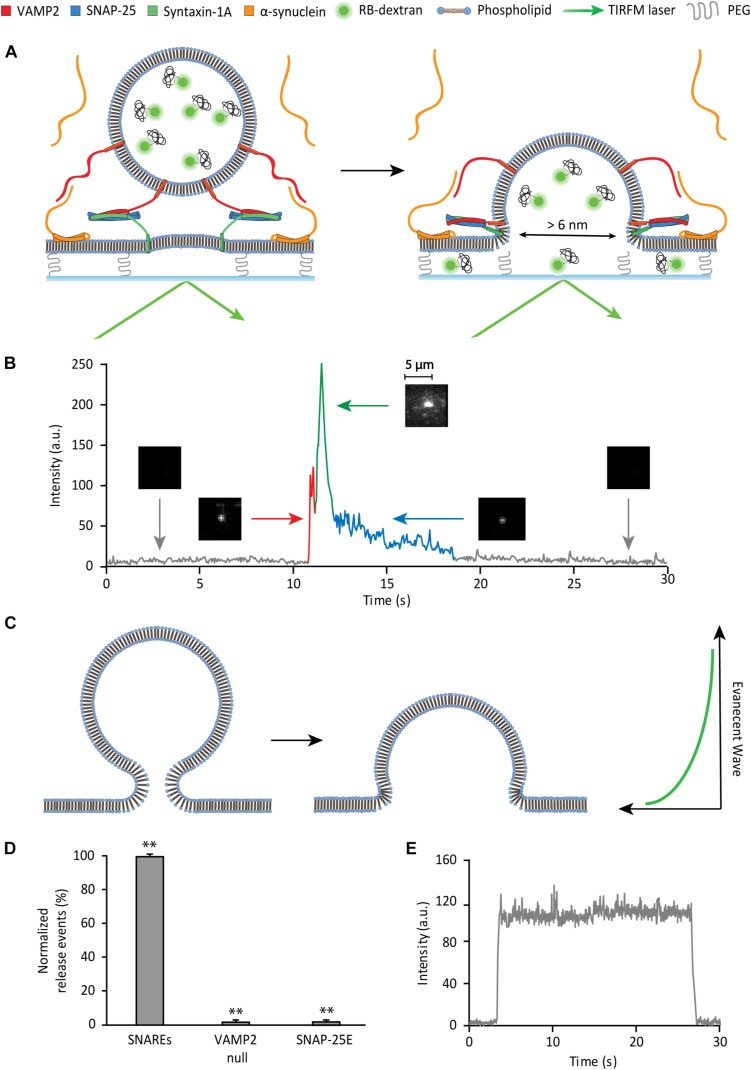
Single vesicle-to-supported bilayer fusion assay to monitor a large fusion pore. **(A)** Schematic of the content release assay. **(B)** Detection of large pore fusion event. Before the injection of vesicles, there is a black background (gray trace). As vesicles are injected and dock to the surface, there is a spike in the fluorescence intensity (red trace) visualized by a fluorescent spot. After a short plateau (red trace), the vesicle develops a large fusion pore, where the fluorescence intensity increases sharply and declines sharply thereafter (green trace). During this period of increase and decline, the vesicle discharges content, which can be visualized by 2D diffusion of fluorophores. As the large pore contracts in size, the internal content escapes slowly, producing a slow decay (blue trace). The 2D diffusion of fluorophores is the criterion that divides the green trace and the blue trace. During the blue trace, 2D diffusion of fluorophores is not observed. This is visualized by a gradual dimming of the fluorescent spot to the black background. **(C)** Hypothetical model of membrane deformation in vesicle fusion. Here, SNARE complexes are not shown for clarity. **(D)** Controls for the SNARE dependence of the large fusion pore. The data are shown as means ± SD. ** *p* < 0.01 by Student’s *t*-test; *n* = 3 independent experiments. **(E)** Fluorescence trace of a non-release event. There is no decay in fluorescence over the span of over 20 s, indicating photobleaching is negligible.

To probe the SNARE-induced large fusion pore, we monitor single vesicle-to-supported bilayer fusion utilizing total internal reflection fluorescence microscopy (TIRFM) (Figure 2A). The supported bilayer contains 5 mole% polyethylene glycol (PEG)-PE that creates a PEG-pillared aqueous gap between the bilayer and the quartz support through which a fluorescent reporter could diffuse. We use Rhodamine B conjugated to 10 kD dextran (RB-dextran) as the fluorescent reporter for the fusion pore. The rationale for using RB-dextran is two-fold. Firstly, the hydrodynamic diameter of the molecules is estimated to be ∼6 nm. Thus, unlike small fluorescent probes, its 2D diffusion within the aqueous gap is predicted to be sufficiently slow to be readily visible with TIRFM, which captures data with millisecond time resolutions. Secondly, RB-dextran is allowed to escape from the vesicle when the fusion pore opens larger than ∼6 nm in diameter, enabling the detection of a large fusion pore.

The supported bilayer is prepared by spontaneous fusion of proteoliposomes, reconstituted with t-SNAREs (lipid-to-protein ratio (L/P) = 2000), onto a clean, hydroxylated quartz surface in the flow cell. The quality and the homogeneity of the supported bilayer are visually inspected under a microscope with a small amount (0.5 ppm) of the lipid dye DiD (1,1′-Dioctadecyl-3,3,3′,3′- Tetramethylindodicarbocyanine). Separa tely, v-SNAREs are reconstituted into liposomes that encapsulate RB-dextran as the internal content (v-vesicle).

To monitor the large fusion pore, the v-vesicles are injected into the flow cell containing the preformed supported bilayer. The formation of the SNARE complexes mediates vesicle docking and fusion. When a v-vesicle docks to the bilayer, a fluorescent spot appears on the imaging surface (red spike in Figure 2B). Subsequently, if a fusion pore greater than 6 nm in diameter is induced, we observe 2D diffusion of fluorophores as RB-dextran is dispersed underneath the supported bilayer (green trace in Figure 2B). The large pore often contracts prior to complete fusion. Pore contraction results in a solid fluorescence spot with a reduced intensity that gradually fades to dark (blue trace in Figure 2B). The slow decrease of fluorescence in this phase most likely indicates the slow leakage of the polymer content via a small fusion pore (please see somewhat different interpretation of the data in Kreutzberger et al., 2019). In fact, Chapman and coworkers recently shown that SNARE complexes alone can sustain prolonged opening of a small fusion pore (Bao et al., 2018).

Surprisingly, in the time trace, we observe that content release causes the initial increase and then, the subsequent decrease of the fluorescence intensity (green trace in Figure 2B). Since the entrapped fluorophore concentration (90 μM) is lower than the critical concentration for self-dequenching, we rule out the possibility that the initial increase of fluorescence is due to self-dequenching. A likely scenario for the increase of the fluorescence intensity is that the internal content of the vesicle moves into the region of the stronger evanescent wave during formation of the large pore (Figure 2C). A similar increase of the fluorescence intensity was observed by Tamm and coworkers during the late stage of fusion of dense core vesicles to the planner bilayer employing the mRuby dye tagged to 36-residue neuropeptide Y (Kreutzberger et al., 2019). They interpreted the data similarly. The data suggests that the larger the size of the fusion pore, the more the vesicle would collapse to the surface, and the higher the fluorescence intensity increase in this phase would be. Thus, the increase of the fluorescence intensity here is likely to reflect the size of the fusion pore. We note, however, that the fluorescence steps from fusion of dense core vesicles are not entirely identical to those from our experiment. In particular, Tamm and coworkers observed a decrease of the fluorescence intensity after the docking plateau between red and green traces in Figure 2B. Such a decrease is not present in our time traces. The difference is most likely due to the fast kinetics in our vesicle-to-supported bilayer fusion.

As controls, to confirm that content release is SNARE-dependent, individual SNARE proteins are omitted or replaced with a disabled mutant in separate assays (Figure 2D). The v-SNARE dependence is tested using v-vesicles without VAMP2. When these vesicles are flowed over the bilayer, there are virtually no content release events. The t-SNARE dependence is evaluated using a SNAP-25 truncation mutant, SNAP-25E that is derived from the product of Botulinum toxin E cleavage that removes 26 residues from the C-terminal SNARE motif. SNAP-25E, which has been shown to impair vesicle docking (Arrio-Dupont et al., 1996), supports no content release events. In both controls, there are non-release events displaying vesicles that transiently dwell on the bilayer, without content release, followed by disengagement from the membrane (Figure 2E). Events with such a fluorescent trace pattern are excluded in the analysis.

### SNAREs Are Not Effective in Driving Formation of the Fusion Pore Larger Than 6 nm

**FIGURE 3:**
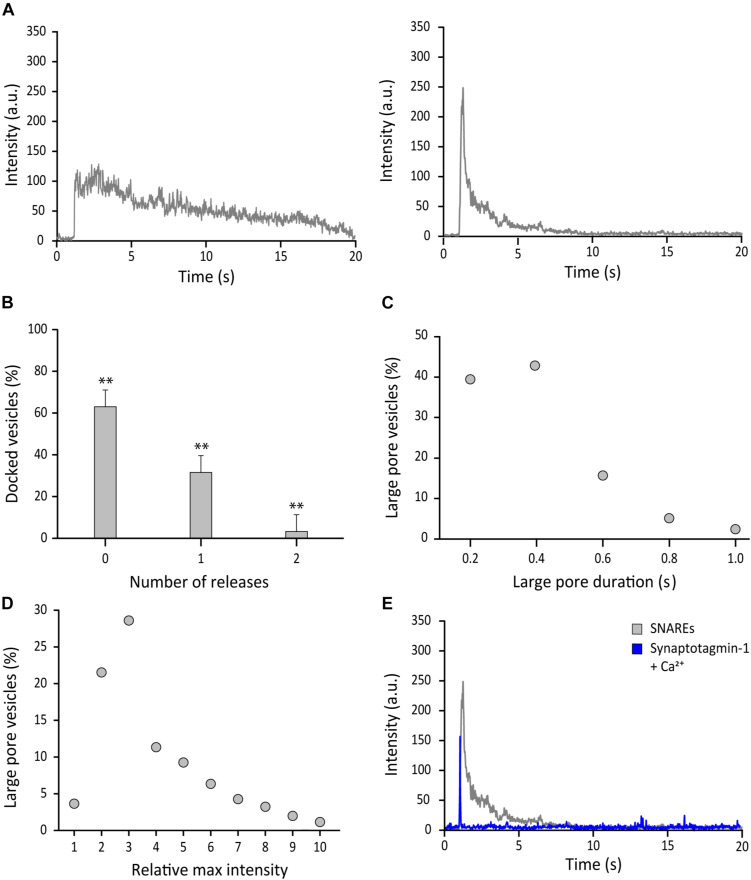
SNAREs are inefficient at driving formation of the fusion pore larger than 6 nm. **(A)** Representative fluorescence time-traces of SNARE mediated fusion. Docked vesicles without apparent release (left) and with release (right) determined by 2D diffusion of fluorophores are shown. **(B)** Distribution of the number of large pore fusion events for individual docked vesicles. Total 4 independent measurements were analyzed for B–D. Error bars represent standard deviations from means. The data are shown as means ± SD. ** *p* < 0.01 by Student’s *t-*test; *n* = 3 independent experiments. **(C)** Distribution of duration of the large fusion pore, which is measured by the green part in Figure 2B. **(D)** Distribution of the relative maximum fluorescence intensity. The relative maximum fluorescence intensity of each event is calculated by dividing the maximum intensity caused by fusion pore expansion by the fluorescence intensity at the moment of vesicle docking. This is necessary because every vesicle has different number of content dyes. **(E)** Representative time traces of the content release event with SNAREs alone (gray) and with synaptotagmin-1 and 500 μM Ca^2+^ (blue). Synaptotagmin 1 and Ca^2+^ were premixed before injection (Kim and Shin, 2017). The molar ratio of VAMP-2 vs. synaptotagmin 1 in the vesicle is 1:1. More than 100 time traces are collected and they all show the similar pattern.

With SNAREs alone, a majority of the vesicles that dock to the surface of the bilayer via SNARE zippering do not open a large pore. Out of the 285 events analyzed, 177 vesicles (62%) do not show a sharp change of fluorescence nor 2D diffusion of fluorophores, indicating that the large fusion pore was not formed. A prototypical fluorescence time trace representing this group shows a spike in fluorescence due to docking, but it is followed by a slow decay of fluorescence to the baseline over several seconds (Figure 3A left).

A minor population was able to open the large pore briefly before contracting. This led to a trace with an initial sharp increase and decrease of fluorescence prior to the slow leakage phase (Figure 3A right). More precisely, 108 out of 285 (38%) vesicles analyzed show the sharp increase then decrease of fluorescence and concurrent 2D diffusion of the fluorophores, reminiscent of the large fusion pore (Figure 3B). We also note that for a small percentage of the docked vesicles (5%), we observe two discrete release events from a single docked vesicle. These events are separated and are not included in the analysis of 285 total events. Thus, with SNAREs only, the probability of the large fusion pore for a single docked vesicle is 38%, indicating that SNARE complex alone is not an efficient driver of fusion pore expansion.

Besides the probability, other important parameters such as the duration and the qualitative pore size can be estimated from the data. For the majority of large pore fusion events, the large pore contracts after the partial release, rarely reaching the full release. The duration of the large fusion pore is defined as the time lapse of the green trace in Figure 2B. The duration distributes between 0.02 s and 1.00 s with an average of 0.28 ± 0.18 s (Figure 3C). On the other hand, the intensity increases due to the flattening (or collapse) of the vesicle during large pore formation provides qualitative estimation of the fusion pore size. The fluorescence intensity increases as much as 10 times with the median at 3 times (Figure 3D), indicating significant flattening (or collapsing) of the vesicle during the large pore fusion event.

It was previously shown that synaptotagmin-1 promotes dilation of the fusion pore (Lai et al., 2013). Thus, as a positive control, we added synaptotagmin-1 together with 500 μM Ca^2+^ concentration (Kim and Shin, 2017). In contrast to the case with SNAREs only, most vesicles show the full content release in a short time span (∼0.02 s) producing a sharply spiked time trace (Figure 3E). Thus, the results show that synaptotagmin-1 with Ca^2+^ is a major stimulator for fusion pore dilation, consistent with previous findings (Lai et al., 2013; Wu et al., 2019).

### αS Promotes the Probability, the Duration, but Not the Size of the Large Fusion Pore

The vesicle-to-supported bilayer fusion assay employing a large polymer cargo provides an opportunity to dissect the effect of the fusion modulators on the formation of the large fusion pore. In Figure 3, we demonstrate that the analysis of individual single vesicle fusion can yield probability, duration, and relative size of the large fusion pore. To uncover the effect of αS on the formation of SNARE-induced large pore, we included 5 μM αS, a typical cellular concentration, in our membrane fusion assay (Hawk et al., 2019). The vesicles are premixed with αS and after 10 min incubation, the mixture is injected into the flow chamber. The flow chamber also was incubated with 5 μM αS before injection.

**FIGURE 4:**
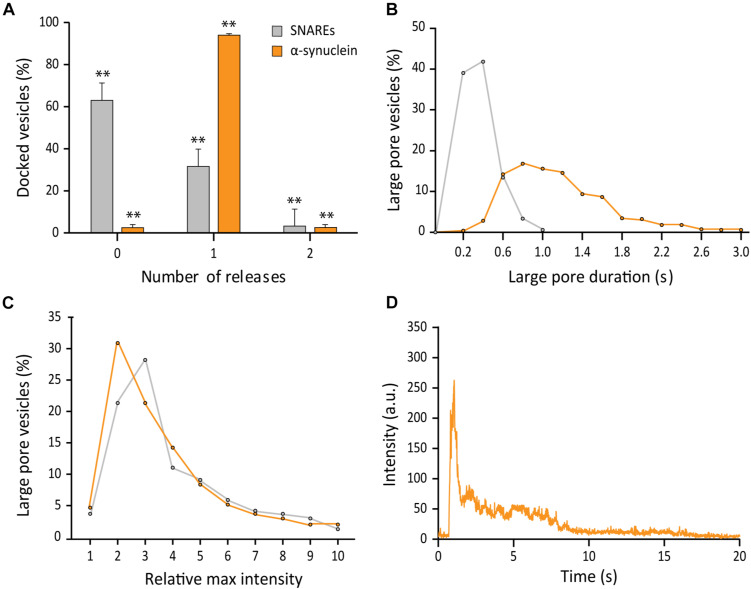
αS promotes probability, duration, but not the size of the large fusion pore. **(A)** Distribution of number of large pore fusion events for individual docked vesicles. For αS, events from 7 independent measurements were analyzed for **A–C**. Error bars represent standard deviations from means. The data are shown as means ± SD. ** *p* < 0.01 by Student’s *t*-test; *n* = 3 independent experiments. **(B)** Distribution of the duration of the large fusion pore. **(C)** Distribution of relative maximum intensities of large pore fusion events. **(D)** Representative fluorescence time trace of a large pore fusion event with αS.

Firstly, out of 691 events analyzed, we found that αS drastically increases the probability of a docked vesicle to form a large fusion pore. As much as 97% of docked vesicles showed the release of RB-dextran, which is in sharp contrast with 38% for SNAREs only (Figure 4A). However, even in the presence αS, vesicles struggled to reach complete release through a large pore prior to contraction. Secondly, with αS, the duration of the large pore is 1.15 ± 0.67 s on average, which is an increase by about factors of 4 compared with the average duration with SNAREs only (Figure 4B). Thirdly, we found that with αS, there is no further increase of the fluorescence intensity at the initial phase of formation of the large fusion pore, indicating that sizes of the large fusion pore remain approximately the same as those of SNAREs only (Figure 4C). This resulted in αS displaying a trace with a longer release phase than the trace of SNAREs alone (Figure 4D). Thus, the results show that αS increases the probability and the duration of the large fusion pore significantly, while the size of the large fusion pore is largely unaffected.

### The SNARE Interaction With αS Does Not Affect Fusion Pore Expansion

Structurally, αS is composed of distinct two parts, the membrane-binding amphipathic region of N-terminal 100 residues and the acidic C-terminal region of 40 residues. The soluble C-terminal region may be functionally important because it interacts with the N-terminal region of v- SNARE, VAMP2. Previously, we have shown that αS can cross-bridge a vesicle to the lipid bilayer by utilizing these two interactions (Lou et al., 2017). It is unclear if such cross-bridging can affect the fusion pore. To test this idea, we generated the truncation mutant αS 1-95 by removing the final 45 amino acids from the C-terminal of αS (Lai et al., 2014; Lou et al., 2017).

**FIGURE 5:**
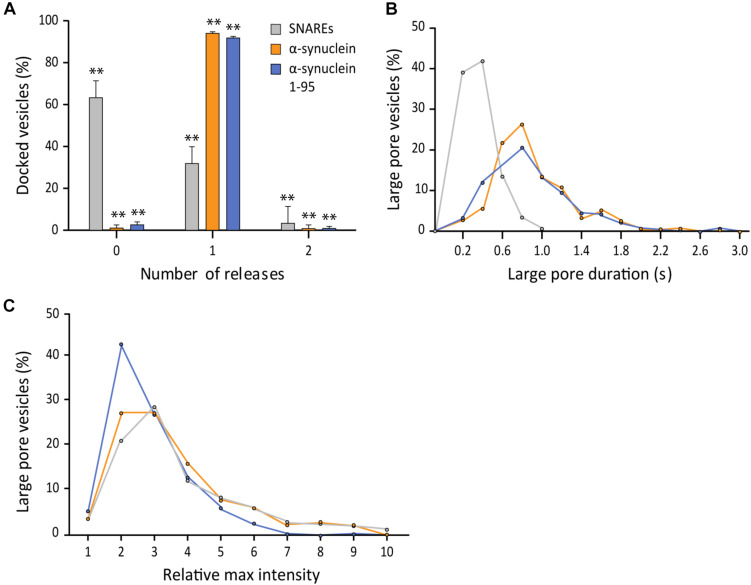
The SNARE interaction with αS does not affect fusion pore expansion. **(A)** Distribution of number of large pore fusion events for individual docked vesicles. For αS 1-95, events from 5 independent measurements were analyzed for **A–C**. Error bars represent standard deviations from means. The data are shown as means ± SD. ** *p* < 0.01 by Student’s *t*-test; *n* = 3 independent experiments. **(B)** Distribution of the duration of the large fusion pore. **(C)** Distribution of relative maximum intensities of large pore fusion events.

This time, we used 200 nM for both wild-type αS and αS 1-95 due to the aggregation of αS 1-95 at the μM concentration range under our experimental conditions. Out of 403 events analyzed, we found that the probability of the large fusion pore for αS 1-95 is similar to that for wild-type αS (99% vs. 97%, respectively) (Figure 5A). However, the average duration of the large pore for αS 1-95 is slightly shorter than that for wild-type αS (0.86 ± 0.50 s vs. 1.15 ± 0.67 s, respectively) (Figure 5B). With αS 1-95, the increase of the fluorescence intensity at the initial phase of large pore formation was somewhat less (2.86 ± 1.53) than the increase for the wild-type (3.38 ± 2.20) (Figure 5C). Thus, although there are some minor variations, our results show that the C-terminal of αS and possibly, its interaction with VAMP2 is not much to do with the stimulation of large fusion pore formation.

### Membrane Binding of αS Plays a Role in Stimulating Fusion Pore Expansion

**FIGURE 6:**
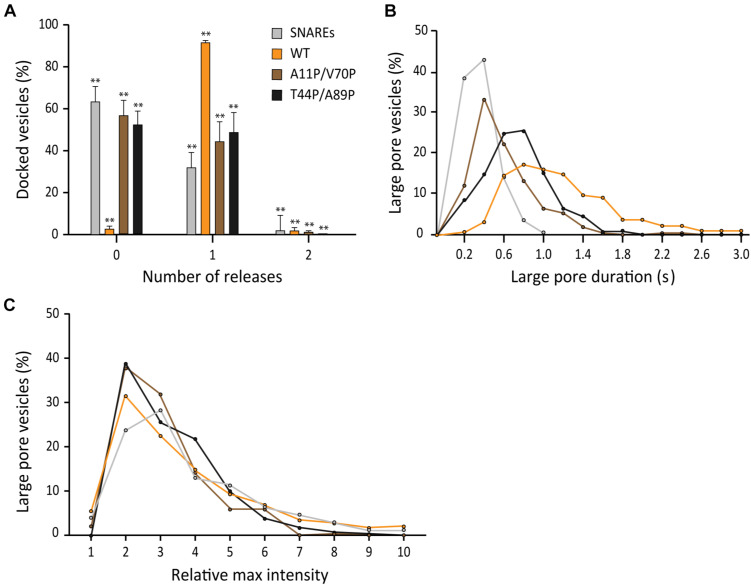
Membrane binding of αS plays a role in stimulating fusion pore expansion. **(A)** Distribution of number of large pore fusion events for individual docked vesicles. For αS A11P/V70P, 124 release events were detected out of 289 total events from 18 independent measurements. For αS T44P/A89P, 98 large pore fusion events were detected out of 209 total events from 19 independent measurements. Error bars represent standard deviations from means. The data are shown as means ± SD. ** *p* < 0.01 by Student’s *t*-test; *n* = 3 independent experiments. **(B)** Distribution of the duration of the large fusion pore. **(C)** Distribution of relative maximum intensities of large pore fusion events.

After learning that the interaction between αS and v-SNARE VAMP2 has minimal effects on the parameters of fusion pore expansion, we asked if membrane binding of αS is the factor that governs its stimulatory role in fusion pore expansion. Recently, Sudhof and coworkers isolated and characterized double proline mutants of αS A11P/V70P and T44P/A89P whose membrane binding activity is significantly impaired (Burré et al., 2015). In parallel to their impaired membrane-binding activity, both double proline mutants showed significantly reduced stimulation of fusion pore expansion compared to that by the wild-type. In the presence of the mutants, the probability to open a large pore reaches only 50%, which is slightly higher than that of SNAREs only, but much lower than 97% in the presence of wild-type αS (Figure 6A). Similarly, the durations of the large fusion pore are longer than those with SNAREs only, but shorter than those in the presence of the wild-type (Figure 6B). Interestingly, it appears that the rank order of the durations follows the rank order of the membrane affinity (Lai et al., 2014), where the wild-type is the first, T44P/A89 the second, and A11P/V70P the third for the both parameters. There was no significant different in pore sizes between the wild-type and the mutations (Figure 6C). Thus, our results show that membrane binding of αS is the major factor that determines its activity of stimulating fusion pore expansion.

### Familial Mutations A30P and A53T of αS Moderately Reduce the Duration of the Large Fusion Pore

Studies of families with a history of Parkinson’s disease have resulted in the identification of several familial mutations (αS A30P, E46K, and A53T) involved in early-onset forms of the disease (Wong and Krainc, 2017). Of those mutations, Edwards and coworkers have shown that A30P and A53T abolish promotion of large cargo release by αS in chromaffin cells (Logan et al., 2017).

**FIGURE 7:**
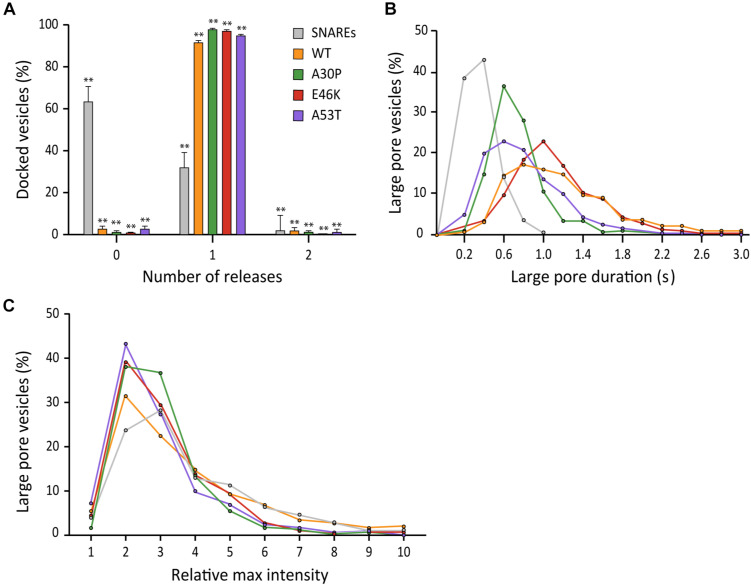
Familial mutations A30P and A53T of αS moderately reduce the duration of the large fusion pore. **(A)** Distribution of docked vesicles vs. number of large pore fusion events. In total for αS A30P, 288 large pore fusion events were detected from 290 total events from 4 independent measurements. For αS E46K, 326 large pore fusion events were detected from 331 total events from 5 independent measurements. For αS A53T, 322 large pore fusion events were detected from 337 total events from 7 independent measurements. Error bars represent standard deviations from means. The data are shown as means ± SD. ** *p* < 0.01 by Student’s *t*-test; *n* = 3 independent experiments. **(B)** Distribution of the duration of the large fusion pore. **(C)** Distribution of relative maximum intensities of large pore fusion events.

To test if their findings are applicable for neuronal SNAREs, we examined three αS point mutants with our single vesicle-to-supported bilayer assay. When compared to wild-type αS, all three mutants have nearly identical capacities to increase the probability of large fusion pore formation with probabilities >90% (Figure 7A). Interestingly, the duration of the large pore is somewhat reduced to 0.61 ± 0.20 s for A30P and 0.69 ± 0.37 s for A53T compared to 1.15 ± 0.67 s for the wild-type (Figure 7B). Meanwhile, the duration for E46K is 1.05 ± 0.42 s, which is similar to that for the wild-type within experimental uncertainty. For the increase of the fluorescence intensity at the initial phase of the release, all familial mutants and the wild-type are statistically similar to each other (Figure 7C). Thus, the results suggest that for all αS familial mutations, the fusion pore can grow as large as the size of the wild-type, but for A30P and A53T, the enlarged pore is not as stable as that of the wild-type.

## Discussion

In this work, we have found, using an *in vitro* single vesicle fusion assay that αS has the capacity to promote the formation of the large fusion pore in SNARE-dependent membrane fusion. Our results are consistent with the findings by Edwards and coworkers (Logan et al., 2017), that αS enhances the release of a large protein cargo in chromaffin cells. For neuronal SNAREs, our data shows that αS increases the probability of individual vesicles to advance to the large fusion pore (diameter larger than 6 nm). Moreover, our results show that the duration of the large fusion pore is also increased by αS significantly. Consistently in neurons, Edwards and coworkers have found that αS delays the closing of the fusion pore for the release of small neurotransmitter, therefore, most likely the small fusion pore. Thus, the results together support the conclusion that αS has the tendency to keep the fusion pore open longer than it is without αS, regardless of the size of the fusion pore. Our results show that SNARE complex alone is only capable of expanding the fusion pore larger than 6 nm in diameter for less than 40% of the docked vesicles. More than 60% of them are not able to reach a pore size sufficiently large to allow release of 6 nm-diameter RB-dextran. Interestingly, we find that the SNARE-induced large pore is transient. In the presence of αS, the probability of large fusion pore formation is increased to nearly 100%.

Regardless of the presence of αS, after some release, the fusion pore contracts back to a very slow release state, which is most likely a small pore stage. Such incomplete dilation leaves residual RB-dextran in the vesicle. This suggests that although SNARE complexes can generate a transient large fusion pore, they are not fully sufficient to drive the complete dilation of the fusion pore. In sharp contrast, fusion pore expansion is fast and ends up with complete decantation of the content in the presence of synaptotagmin-1 and Ca^2+^. Thus, we speculate that synaptotagmin-1 and Ca^2+^ are the determining factors that drive fusion pore expansion into completion.

We speculate that the stimulating effects of synaptotagmin-1 and Ca^2+^ and that of αS are additive because synaptotagmin-1 and αS would not likely compete with each other for membrane binding. Ideally, this prediction could be tested with a similar *in vitro* vesicle fusion assay to the current one, but with a faster time resolution. The current method is limited with the time resolution of 20 msec, which is too slow to resolve the kinetics of fusion pore expansion in the presence of synaptotagmin-1 and Ca^2+^.

On a molecular level, αS is known for the interaction with v-SNARE VAMP2 as well as its interaction with the negatively charged membrane. The former is mediated by the binding between the C-terminal end of αS and the N-terminal region of VAMP2. However, our results show that this specific interaction has little to do with the probability, the duration, and the size of the large fusion pore. When the VAMP2-binding C-terminal region of αS was eliminated the three parameters remain virtually the same as that of the wild-type. Alternatively, the latter is mediated by the affinity of the N-terminal amphipathic region to the acidic lipids. Our data unambiguously finds that αS’s membrane-binding property plays a major role in fusion pore dilation. We speculate that αS binds to the membrane and adapts to the diverse architecture of the fusion pore, which stabilizes the membrane curvature (Trexler and Rhoades, 2009). For example, Trexler and Rhoades have shown that αS has preferential binding to the positive membrane curvature. The positive curvature surrounding the fusion pore could be stabilized by αS binding.

In chromaffin cells, Edwards and coworkers found that the familial mutations A30P and A53T abolish the ability of αS to promote large cargo release. Intriguingly, we find that, although the differences are small, these same familial mutations are not as efficient as the wild-type in providing the stability of the enlarged fusion pore (Figure 7B). However, it is not clear if such a small effect is relevant to the early onset of the Parkinson’s disease, warranting further investigation.

In this work, we have demonstrated that the single vesicle to supported bilayer fusion method is highly effective in dissecting the regulation of fusion pore expansion by αS. However, there are some weaknesses of the method. Although we add certain amounts of proteins, we have no way of estimating the exact number of molecules at the fusion site. We point out the new nanodisc approach, developed by other groups (Bao et al., 2018; Wu et al., 2019), with which one can control the number of proteins. We believe that the two methods will serve complimentarily.

In summary, elucidating the mechanism by which αS regulates SNARE-dependent membrane fusion is of great general interest. In this work, using the *in vitro* single vesicle-to-supported bilayer fusion assay employing 10 kD RB-dextran, we demonstrate that the SNARE complex could drive the enlargement of the fusion pore greater than 6 nm in diameter, but it collapses without progressing toward full dilation. However, in the presence of αS, more vesicles reach states of the sustained large fusion pore. Our results suggest that membrane binding of αS is responsible for the stimulating activity of fusion pore expansion.

## Materials and Methods

### Plasmid Constructs and Site-Directed Mutagenesis

DNA sequences encoding SNAP-25 (amino acids 1-206), SNAP-25E (amino acids 1-180), syntaxin-1A (amino acids 1-288), VAMP2 (1-116), αS (amino acids 1-140) including all mutations, and αS 1-95 (amino acids 1-95) are inserted into the pGEX-KG vector as N-terminal glutathione S-transferase (GST) fusion proteins. Native cysteines are replaced by alanines for all the sequences. DNA sequences are confirmed by the Iowa State University DNA Sequencing Facility.

### Protein Expression and Purification

N-terminal SNARE GST fusion proteins (SNAP-25, SNAP-25E, syntaxin-1A, and VAMP2) are expressed in *Escherichia coli* BL21 (DE3) competent cells. Cells are grown at 37°C in LB medium with ampicillin (100 μg/mL) until the absorbance at 600 nm reaches 0.6–0.8, and induced by the addition of IPTG (isopropyl β-D-thiogalactopyranoside, 0.3 mM final concentration) to express the protein overnight at 16°C. Cells are pelleted and resuspended in a wash solution [497 mM NaCl, 2.7 mM KCl, 10 mM Na_2_HPO_4_, 1.8 mM KH_2_PO_4_, pH 7.4, (4 g/L Triton-X 100 added for the membrane proteins, VAMP2 and syntaxin-1A)] with final concentrations of 1 mM AEBSF [4-(2-aminoethyl) benzenesulfonyl fluoride and 4 mM DTT. Cells are lysed by homogenization and centrifuged to separate the supernatant from the pellet. The supernatant is collected and mixed with reduced glutathione resin in a batch purification method. After incubation, the protein is purified by washing the resin with wash solution. After washing, the resin is equilibrated into an elution buffer [137 mM NaCl, 2.7 mM KCl, 10 mM Na_2_HPO_4_, 1.8 mM KH_2_PO_4_, pH 7.4, (0.8% octyl-beta-glucoside (OG) was added for the membrane proteins)]. Proteins were eluted by cleavage with 30 U of thrombin at 4°C for 16 h. The protein was stored at −80°C with 15% glycerol. αS (wild type, mutants, and truncated variants) are expressed and purified in the same manner as detailed above, but have an additional step.

The elution was further purified using FPLC employing a home-made size exclusion column packed with toyopearl HW-50F in a 2.5 cm × 60 cm Chromaflex column. PBS (137 mM NaCl, 2.7 mM KCl, 10 mM Na_2_HPO_4_, 1.8 mM KH_2_PO_4_, pH 7.4) was used as the mobile phase. 5 mL fractions were collected and samples of each fraction were run on a 15%-SDS-PAGE gel to identify fractions that lacked higher molecular weight species. Pure fractions were combined, concentrated, and stored at −80°C with 15% glycerol.

### Lipid Preparation

The lipids used to form the supported-bilayer (t-lipids) are made using a mixture of POPC (1-palmitoyl-2-dioleoyl-sn-glycero-3-phosphatidylcholine), DOPS (1,2-dioleoyl -sn-glycero-3-phosphatidylserine), PIP2 (phosphatidylinositol 4,5-bisphosphate), and PEG2000-PE {1,2-dipalmitoyl-sn-glycero-3-phosphoethanolamine-N-[methoxy(polyethylene glycol)-2000]} in chloroform at a molar ratio of 78:15:2:5. The lipid mixture is first dried under an air stream, then dried further in a vacuum overnight. The t-lipids are resuspended in HEPES-OG buffer (25 mM HEPES/KOH, 150 mM KCl, 1% β-OG, pH 7.4).

The lipids used to form liposome for v-SNARE VAMP2 reconstitution (v-lipids) are made using a mixture of POPC, DOPS, and cholesterol in chloroform at a molar ratio of 75:5:20. The v-lipids are resuspended in HEPES with 90 μM Rhodamine B conjugated to 10 kD dextran (RB-dextran) before 10 flash freeze-thaw cycles, alternating between liquid nitrogen and boiling water. Unilamellar vesicles were prepared by extrusion through 100 nm diameter polycarbonate filters to make v-liposomes.

### SNARE Reconstitution

For the supported bilayer, syntaxin-1A and SNAP-25 are premixed in a molar ratio of 1:1.5, and the mixture incubated at room temperature to form the t-SNARE complex. The t-lipids are added to the t-SNARE complex at a lipid:syntaxin-1A ratio of 2000:1. The mixture is diluted 3-fold using HEPES buffer to reduce detergent concentration and insert the t-SNARE complex into the t-lipids. The mixture is then dialyzed overnight at 4°C in 2L of HEPES containing Bio-Beads^TM^ SM-2 Resin to remove all detergent.

For v-vesicles, v-liposomes are mixed with VAMP2 at a lipid-to-protein ratio of 200:1. The mixture is diluted and dialyzed in the same manner as described above while ensuring that concentration of RB-dextran is constant at ∼90 μM. Vesicles prepared with this method were found to be ∼ 90 nm ± 10 nm in diameter with few small unilamellar vesicles (SUV) when examined with transmission electron microscopy (Yoon et al., 2006).

### Vesicle-to-Supported Bilayer Fusion Content-Release Assay

A quartz slide and a glass cover slip are cleaned and hydroxylated by boiling in a piranha solution (1:1 mixture of concentrated sulfuric acid and 30% hydrogen peroxide) for 15 min. Afterward, the slide and cover slip are thoroughly rinsed with deionized H_2_O and placed in a cleaning sonicator for 30 min to remove residual acid. The slide and coverslip are then dried and assembled to generate several microfluidic chambers separated by double sided Scotch tape. The chambers are filled with t-bilayer prepared from the overnight dialysis. The t-bilayer formed on the quartz surface for 2 h at 37°C. The excess liposomes/protein mixture was washed out with HEPES and replaced with 5 μM αS.

The microfluidic chambers are then placed on the imaging stand of our microscope. Imaging oil was put on the prism of our prism-type TIRFM, and then the prism was lowered onto the quartz slide. The incident angle of the exciting laser (532 nm) was adjusted and we initiated real-time movie acquisition with an imaging area of 110 × 110 μm using 20 ms time resolution. The viewing area is divided into the 512 × 512 pixels and the data is stored in the 512 × 512 arrays. To perform the fusion assay, we injected the v-vesicles from dialysis with 5 μM αS into the microfluidic chamber at a rate of 50 μl/min. The sample to be injected into the flow cell has 250 nM of v-vesicles (total lipid concentration) encapsulating ∼90 μM of RB-dextran. The sample contains 3.75 nM of RB-dextran in the bulk solution which does not affect our measurements. We collected 60 s videos for each microfluidic chamber and analyzed fusion events using our custom-built analysis software.

### Data Analysis

Fluorescence of RB-dextran from the content vesicles is monitored to determine content release from fusion events using in-house MATLAB^®^ 2019 (a) analysis software. Each recording is analyzed frame by frame based on both visual determination and fluorescence trace pattern analysis.

The fluorescence intensities shown in Figure 2 are calculated by summing up those in 5 × 5 pixels surrounding the central pixel with the brightest light intensity.

Large pore content release is indicated when a vesicle immobilized and fused on the surface displays 2D diffusion of the fluorophore. The corresponding fluorescence trace shows a large spike in fluorescence followed by a sharp decrease within less than 2 s. Events that did not form a large pore are indicated when a vesicle immobilized on the bilayer with no visible 2D diffusion of the fluorophore. The corresponding fluorescence trace shows a large spike in fluorescence followed by a slow decay to baseline over several seconds. Non-release events are when a vesicle became immobilized on the surface and disengaged after several seconds without any visible release. The fluorescence trace of a non-release event that contained a sharp increase in fluorescence, did not decay over several seconds during a plateau period, and then sharply declined to baseline. Non-release events were not included in the data analysis. This lack of fluorescence decay also indicates that photobleaching is not observable in the time scales we are measuring.

The selected traces corresponding to large pore content release events are background-corrected by fitting the minimum baseline for all traces from a single recording with a polynomial and then subtracting the polynomial from all the traces. The number of content release events are manually counted. The duration of release was quantified as the time from the beginning to end of large pore content release which is determined by the period of apparent 2D diffusion of the fluorophore.

## Data Availability Statement

The raw data supporting the conclusions of this article will be made available by the authors, without undue reservation.

## Author Contributions

RK contributed to the protein purification, data collection, and analysis of the experiments along with drafting the manuscript. BH contributed to developing the data analysis program. T-MK and JP contributed to the protein purification and data collection. GY and NL provided the plasmids for the αS truncation studies. Y-KS was responsible for the directing the research and manuscript drafting. All authors contributed to the article and approved the submitted version.

## Conflict of Interest

The authors declare that the research was conducted in the absence of any commercial or financial relationships that could be construed as a potential conflict of interest.
